# Natural Fiber-Reinforced Polycaprolactone Green and Hybrid Biocomposites for Various Advanced Applications

**DOI:** 10.3390/polym14010182

**Published:** 2022-01-03

**Authors:** R. A. Ilyas, M. Y. M. Zuhri, Mohd Nor Faiz Norrrahim, Muhammad Syukri Mohamad Misenan, Mohd Azwan Jenol, Sani Amril Samsudin, N. M. Nurazzi, M. R. M. Asyraf, A. B. M. Supian, Sneh Punia Bangar, R. Nadlene, Shubham Sharma, Abdoulhdi A. Borhana Omran

**Affiliations:** 1 School of Chemical and Energy Engineering, Faculty of Engineering, Universiti Teknologi Malaysia (UTM), Johor Bahru 81310, Johor, Malaysia; saniamril@utm.my; 2 Centre for Advanced Composite Materials (CACM), Universiti Teknologi Malaysia (UTM), Johor Bahru 81310, Johor, Malaysia; 3 Institute of Tropical Forestry and Forest Products (INTROP), Universiti Putra Malaysia (UPM), Serdang 43400, Selangor Darul Ehsan, Malaysia; mohdsupian7779@gmail.com; 4 Advanced Engineering Materials and Composites Research Centre (AEMC), Department of Mechanical and Manufacturing Engineering, Universiti Putra Malaysia (UPM), Serdang 43400, Selangor, Malaysia; 5 Research Center for Chemical Defence, Universiti Pertahanan Nasional Malaysia, Kem Sungai Besi, Kuala Lumpur 57000, Malaysia; faiznorrrahim@gmail.com; 6 Department of Chemistry, College of Arts and Science, Davutpasa Campus, Yildiz Technical University, Esenler, Istanbul 34220, Turkey; syukrimisenan@gmail.com; 7 Department of Bioprocess Technology, Faculty of Biotechnology and Biomolecular Sciences, Universiti Putra Malaysia (UPM), Serdang 43400, Selangor, Malaysia; azwan.jenol@gmail.com; 8 Centre for Defence Foundation Studies, Universiti Pertahanan Nasional Malaysia (UPNM), Kem Perdana Sungai Besi, Kuala Lumpur 57000, Malaysia; mohd.nurazzi@gmail.com; 9 Institute of Energy Infrastructure, Universiti Tenaga Nasional, Jalan IKRAM-UNITEN, Kajang 43000, Selangor, Malaysia; asyrafriz96@gmail.com; 10 Department of Food, Nutrition and Packaging Sciences, Clemson University, Clemson, SC 29631, USA; snehpunia69@gmail.com; 11 Fakulti Kejuruteraan Mekanikal, Universiti Teknikal Malaysia Melaka, Melaka 76100, Malaysia; nadlene@utem.edu.my; 12 Department of Mechanical Engineering, IK Gujral Punjab Technical University, Jalandhar 144001, India; shubham543sharma@gmail.com; 13 Department of Mechanical Engineering, College of Engineering, Universiti Tenaga Nasional, Jalan Ikram-Uniten, Kajang 43000, Selangor, Malaysia; amhmad@uniten.edu.my; 14 Department of Mechanical Engineering, College of Engineering Science & Technology, Sebha University, Sabha 00218, Libya

**Keywords:** polycaprolactone, green biocomposites, hybrid biocomposites, mechanical properties, thermal properties

## Abstract

Recent developments within the topic of biomaterials has taken hold of researchers due to the mounting concern of current environmental pollution as well as scarcity resources. Amongst all compatible biomaterials, polycaprolactone (PCL) is deemed to be a great potential biomaterial, especially to the tissue engineering sector, due to its advantages, including its biocompatibility and low bioactivity exhibition. The commercialization of PCL is deemed as infant technology despite of all its advantages. This contributed to the disadvantages of PCL, including expensive, toxic, and complex. Therefore, the shift towards the utilization of PCL as an alternative biomaterial in the development of biocomposites has been exponentially increased in recent years. PCL-based biocomposites are unique and versatile technology equipped with several importance features. In addition, the understanding on the properties of PCL and its blend is vital as it is influenced by the application of biocomposites. The superior characteristics of PCL-based green and hybrid biocomposites has expanded their applications, such as in the biomedical field, as well as in tissue engineering and medical implants. Thus, this review is aimed to critically discuss the characteristics of PCL-based biocomposites, which cover each mechanical and thermal properties and their importance towards several applications. The emergence of nanomaterials as reinforcement agent in PCL-based biocomposites was also a tackled issue within this review. On the whole, recent developments of PCL as a potential biomaterial in recent applications is reviewed.

## 1. Introduction

Due to the increasing production of waste every day, landfill spaces have become more scarce and have a reduced capacity to intake more waste [1]. Hence, waste disposal is becoming a more problematic task. Composting and incineration are advised as ways to reduce reliance on landfills, and they are becoming increasingly essential [2]. Thus, to take advantage of this emphasis on waste conversion, degradable polymers that are tailored for various methods of waste management are required. Biopolymers, often known as biodegradable plastics, are polymeric materials that degrade, in part, through the metabolism of naturally existing microorganisms via aerobic and anaerobic processes [2,3,4,5,6]. Biodegradation causes the polymers to break or disintegrate under the right conditions of moisture, temperature, and oxygen availability, leaving no hazardous or environmentally detrimental behind [7].

Based on the origin of the raw ingredients and the manufacturing procedures, biopolymers can be categorized into several categories, including natural biopolymers and synthetic biodegradable polymers. Their characteristics are identified and applied widely in medical fields, such as implants, drug delivery, antimicrobial material, sutures and surgeries, among other things [4,5,8,9,10,11]. The example of biopolymer is listed in Table 1 [12].

**Figure 1:**
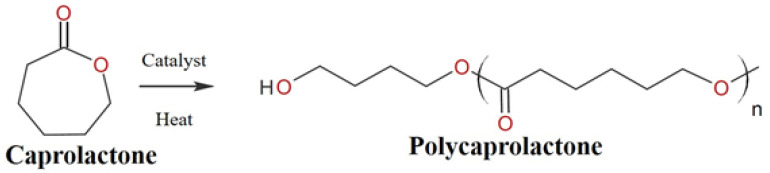
Synthesis of PCL.

Among several types of biopolymers, polycaprolactone (PCL) has received a lot of attention due to its several advantages, such as its biodegradability, high strength, and biocompatibility. It can withstand water, oil, solvents, and chlorine. PCL is a semicrystalline ester polymer that is derived from ring-opening polymerization of ε-caprolactone monomers, as shown in Figure 1. PCL consists of a glass transition temperature (T_g_) of around 60 °C and a melting point ranging between 59–64 °C, dictated by the crystalline nature of PCL which enables easy formability at relatively low temperatures [13].

In contrast, the average molecular weight of PCL samples can range from 3000 to 90,000 g/mol, and they can be classified based on their molecular weight. With increasing molecular weight, its crystallinity decreases. High solubility, a low melting point, and exceptional blend compatibility have sparked a lot of interest in its potential biomedical applications [14]. Table 2 summarizes the effect of PCL molecular weight on their properties [15].

PCL also shows great electrospinning properties, as it can be spun into fibers at temperatures around 200 °C, without undergoing thermal degradation. Since PCL is a synthetic material, it is possible to achieve high material purity. PCL also has relatively long biodegradable time. According to the literature, PCL can biodegrade in a few months to many years, depending on its molecular weight, degree of crystallinity, shape, porosity, sample thickness, and the surrounding environment [16], unlike traditional plastics, such as polypropylene (PP) and polyethylene (PE), which take hundreds or even thousands of years to fully decay.

Recently, due to the environmental concerns, as well as energy and cost considerations, increased research efforts have been directed to the creation of bio-based materials. Bio-based polymers, which minimize reliance on petrochemical-based synthetic polymers, contribute significantly to global environmental sustainability. Recently, Gokhan et al. (2020) [17] has synthesized bio-based PCL from soybean oil-derived polyol via ring-opening polymerization. He found that the soybean-based PCL containing higher PCL molar ratio has poorer biodegradability but higher hydrophobicity and thermal characteristics compared to the others. Nonetheless, biobased polymers possess several weaknesses, including inferior mechanical properties, insufficient heat tolerance, and high moisture sensitivity relative to petroleum-derived polymers [18].

As mentioned in a previous statement, this polymer is well known for being biodegradable and have notably lower degradation. Due to its slow degradation rate, PCL is often used as a material in drug delivery devices that is active for a long time, with a period of over one year [8]. The material is also noted to be potentially used as a bioscaffold [19,20]. In terms of its biodegradability, permeability, and inability to establish an acidic environment, PCL is a better polymer than PLA or PGA in terms of possible biomedical uses [21]. Furthermore, because of the low rate of PCL degradation compared to PLA and PGA, it can be utilized to release medications over longer periods of time, even a year. The hydrolytic cleavage of ester groups is involved in PCL degradation. It is easy to predict that inserting different co-monomer units in the PCL chain will result in diverse properties, including changes in biodegradability, allowing for biodegradability control through targeted introduction of such groups [22]. Manivasagam et al. (2019) [23] have mentioned the application of PCL in dentistry by modifying the material structure with other materials (such as hydrophilic polyethylene glycol (PEG) and ceramic) and introducing co-polymers (such as polylactic acid (PLA) or polyglycolic acid (PGA)). The pKa of the degradation products; the primary mechanisms of the degradation; and the in vivo degradation rate of PCL, PLA, and PGA are summarized in Table 3.

Unfortunately, because PCL manufacture is both complex and expensive, its wider commercialization has been limited. In addition, the material adheres poorly to cells due to its hydrophobic surface. PCL solvents are also known to be toxic, which can potentially harm human beings. The relatively low melting point is also demonstrates another drawback, since it hinders the material from being applied at higher temperatures [25,26,27]. Table 4 shows the summary of advantages and disadvantages of PCL.

Nonetheless, those limitations of PCL can potentially be overcome by the use of PCL-based biocomposites. Extensive efforts have been made over the last decade in the development of PCL-based biocomposites. The properties of PCL can be improved by blending with other polymers or fibres, allowing more people to benefit from its excellent properties [28,29,30,31]. Mechanical and thermal properties are the most influential, affected by the addition of fillers to the PCL [32,33,34]. Several studies have shown that the PCL-based biocomposites can be benefited for several applications, especially in the biomedical field [26,35,36,37,38,39].

Therefore, this review opens up the potential for PCL to be used as a matrix along with natural fibers to form biocomposites. There are two types of biocomposites, i.e., green biocomposites and hybrid biocomposite, which are discussed. Moreover, this review intends to discuss the effect on mechanical and thermal properties of PCL-based biocomposites. Moreover, the potential application of blends of PCL with other materials is also investigated.

## 2. Overview on Natural Fiber

Humans have used natural fibers since the beginning of time, as shown by the discovery of wool and dyed flax 36,000 years ago in Georgia [40]. Natural fibers are derived from fruits, herbs, and animals. Among the engineering materials, natural fibers are used in the additive manufacturing process to create sustainable biocomposite structures [41,42]. Due to their diverse properties, such as their good mechanical behavior, low cost, eco-friendly nature, and biodegradability, they are gaining attention among scientists [43,44,45].

Additionally, natural fibers can be composed of a variety of textures, allowing them to be used in textile additive manufacturing. For example, common natural fibers, such as cotton, can absorb sweat effectively. Soft and lightweight cotton fibers can be manufactured in any size or shape, making them suitable for use with additive manufacturing techniques [46]. In contrast, natural fiber biocomposites are seen as a possible advancement in the field of material science because they are lighter and less costly than synthetic fiber composites. Furthermore, natural fibers are also biodegradable, unlike synthetic fibers, drawing the interest of environmentalists in these challenging circumstances [47].

### 2.1. Types of Natural Fiber

**Figure 2:**
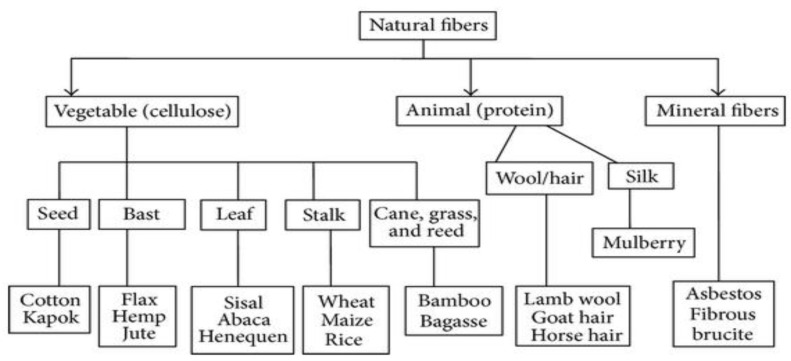
Type of natural fibers.

Natural fibers are those that are produced from plants or animals. It has a number of advantages over synthetic fibers, including their abundance, availability, and low cost [48,49]. These materials possess promising potential for a wide range of industries, including the biomedical, packaging, adsorption, aerospace, textile, food, and automotive industries [50,51,52,53,54,55,56,57,58,59,60,61,62,63,64]. There are three main classes depending on their sources, including vegetables (cellulose), animal, and mineral fibers. Figure 2 shows the types of natural fibers. Plant fibers are the most widely acknowledged fiber within industry and the most thoroughly studied by researchers. This is owing to the short growth period, renewability, and broader availability [65,66,67,68].

### 2.2. Chemical Composition of Green Fibers

The constituents of plant fibers include cellulose, lignin, hemicellulose, pectin, waxes, and water-soluble substances [69,70,71]. The presence of cellulose, which is hydrophilic in nature, affects the interfacial bonding between the polymer matrix and the fibers, since the matrix is hydrophobic [72,73]. These compositions, in turn, will affect the mechanical properties of the fibers [74]. Table 5 shows composition of cellulose, hemicellulose, lignin, and pectin in selected fibers [75].

### 2.3. Mechanical Properties of Natural Fibers

The mechanical properties depend on the chemical composition of the single fibre grooving (climate, soil feature, aging conditions, etc.) and processing method conditions [76]. Usually, the high cellulose content of lignocellulosic fibers is a significant contributor to their excellent tensile properties [77,78]. The mechanical properties of natural fibers can greatly affect the performance of the biocomposites [79,80]. Table 6 summarizes the mechanical properties of natural fibers from various sources [81].

### 2.4. Pre-Treatment of Natural Fibers

Natural fibers have some disadvantages, such as high water absorption and thermal instability. The use of untreated natural fibers usually show decreased strength and modulus when the fibre volume fraction is increased [5,79]. These problems can be overcome by treating the natural fibers [58,82,83,84,85]. Pre-treatment can be carried out either by mechanical, chemical, and biological methods, or a combination of all of them. Chemical pre-treatment is the common method used to pretreat the lignocellulosic biomass in order to completely or partially remove the components [86,87,88]. The main goal of the pre-treatment is to alter or remove structural and compositional impediments to increase yields of intended cellulose products [89]. For example, the presence of hemicellulose and lignin in the lignocellulosic materials result in the ineffective stress transfer between the fibre and matrix, due to lower adhesion. Besides that, the presence of too high of hemicellulose content will affect the degree of crystallinity of fibre, leading to poor mechanical properties of polymer composite obtained [90]. It is necessary to bring a hydrophobic nature to the fibres by suitable chemical treatments in order to develop biocomposites with improved mechanical properties. Therefore, pre-treatment of fibres is important to achieve one or more of the following objectives [91,92]:

- Removal of undesirable fibre constituents;
- Roughening of the fibre surface;
- Separation of individual fibre from their bundles;
- Modification of the chemical nature of the fibre surface;
- Reduction in the hydrophilicity of the fibre;
- Cost-effectiveness;
- Avoidance of degradation or loss of carbohydrate.

Pre-treatment of natural fibers can also strengthen the interaction between the fibers and the polymer matrix [59,93,94]. It increases the interfacial interaction between the matrix and the fibers by lowering the number of OH functional groups on the fibers surface while also lowering the surface roughness. Some chemical compounds, such as silane, acetic acid, sodium hydroxide, acrylic acid, isocyanates, malleated coupling agents, potassium permanganate, peroxide, and others, serve as coupling agents to form a strong interface bond between reinforcement and matrix. Thus, this minimizes the moisture absorption potential. Additionally, the alkalization process is used to physically pretreat natural fibers. This approach improves fiber mechanical properties and leads to stronger fiber interlock with the polymer matrix [95].

Meanwhile, pre-treatment of natural fibers also isolate the nanocellulose [51,96]. Nanocellulose has emerged as a new class of biobased nanofiller material to enhance the performance of biocomposites, including PCL-based biocomposites. Nanocellulose in uniform in diameter and has excellent nanofibrillar morphology [10,97,98]. Nanocellulose can be categorized into cellulose nanofiber (CNF), cellulose nanocrystals (CNC), and bacterial nanocellulose (BNC), depending on their origin and the pre-treatment approach used [60,61,99,100]. The advantages of using nanocellulose, as compared to cellulose in biocomposites, include its higher surface area, aspect ratio, and Young’s modulus. Usually, a small amount of nanocellulose is required to enhance the properties of the biocomposites [63,64,101,102].

## 3. Constituents and Types of Biocomposites

There are several different types of biocomposites used today. Biocomposites can be described as a material composed of two or more distinct constituent materials where one of them is naturally derived [57,63,103]. This combination helps to yield a new material with an improved performance compared to individual neat materials. Biocomposites can be classified based on three classes of reinforcement and polymer materials:

- Totally renewable composites, whereby both the matrix and reinforcement are from renewable resources;
- Partly renewable composites, whereby the matrix is obtained from renewable resources and reinforced with a synthetic material;
- Partly renewable composites, whereby the synthetic matrix is reinforced with natural bio polymers.

**Figure 3:**
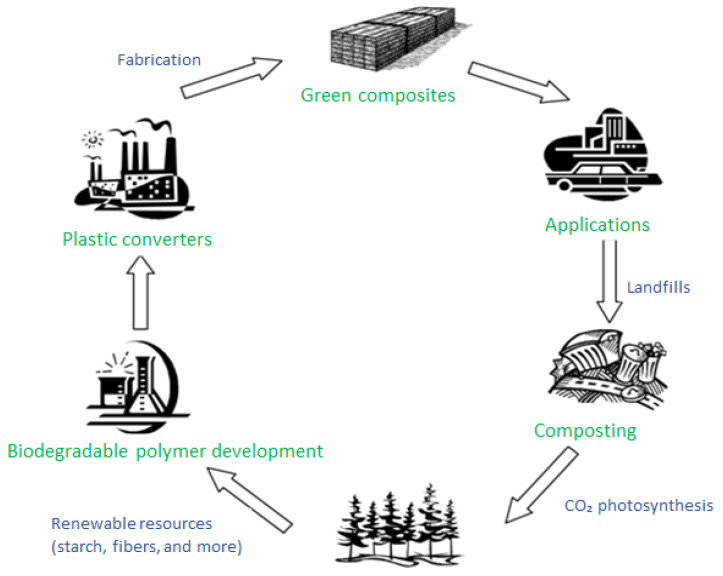
Life cycle of biocomposites.

Figure 3 shows the life cycle of biocomposites. Consequently, biocomposites are a biodegradable and eco-friendly, and can be completely resolved into H_2_O and CO_2_ through microorganism’s degradation in the soil or through an incineration process without causing the emissions of toxic gases [104].

In this review, two types of biocomposites were evaluated: green biocomposites and hybrid biocomposites. Green biocomposites are known for their combination of biodegradable resins with other polymers [105]. Because of their degradable and sustainable properties, they are called green composites, as they can be easily disposed without harming the environment. Ecological concerns, such recyclability and environmental safety, have resulted in renewed interest in natural materials. In recent years, a great interest in research on more environmentally friendly materials derived from non-renewable sources has gained the attention among researchers whose primary aim is to raise public awareness of environmental sustainability [106,107]. Green biocomposites are economical and are easy to decompose. In order to reduce our dependence on fossil fuels, there are more alternatives to develop bio-based resources, such as industrial goods, wood and wood wastes, and other advanced materials. In recent years, low-density natural fibers have been used to reduce the use of expensive glass and carbon fiber, and to reduce the weight of cars. Additionally, natural fibers are less expensive, and have high thermal insulation and biodegradability. Nowadays, by using different type of natural fibers, the green biocomposites have been advanced with enhanced mechanical properties by the researchers. The properties of the natural fibers used for reinforcement can affect the performance of green composites [108]. Herrmann et al. initially reported a study of the fabrication of green composites. They developed a coir–fiber-reinforced polyhydroxybutyrate-co-volerate (PHBV) resin composite and investigated its mechanical properties [109].

Meanwhile, hybrid biocomposites are defined as fabricated materials with two or more fibers inside a single matrix. Hybridization offers new opportunities to broaden the function of the composite materials, particularly in advanced applications by improving the toughness or impact resistance [110]. In this case, PCL refers to a composite which consists of PCL matrix along with two or more fibers within it. Compared to a traditional composite, a hybrid composite offers enhanced properties and advantages. Hybrid composites materials formed by combining two or more different types of fibres in a common matrix are gaining popularity because they provide a variety of properties that cannot be accomplished with a single form of reinforcement. Simultaneously, careful selection of reinforcing fibres can dramatically reduce material costs. Several researchers have independently observed the “hybrid effect” using various types of fibres and reinforcement configurations (such as collimated fibres and fabrics). As a result, it is most likely a real impact rather than a test artifact. Composite failure under tensile load is a complicated, statistical process involving fibre power, matrix, and interfacial properties [111].

## 4. Polycaprolactone-Based Green Biocomposites

Many studies and scientific projects focus on green biocomposites. Green biocomposites integrate the beneficial properties of the individual constituents. Due to the production of materials with good properties and materials which are easily produced and processed, the market for these biocomposites appears to be promising and attainable for growth in the near future [45,112,113]. Biopolymers reinforced with natural fibers greatly improved the mechanical and thermal properties of green biocomposites [114,115,116]. Therefore, several mechanical and thermal properties of PCL-based green biocomposites are discussed in this section.

### 4.1. Mechanical Properties of Polycaprolactone-Based Green Biocomposites

For improved mechanical properties, PCL can be added with stiffer materials and is easily processed by traditional melting techniques due to its low melting temperature. PCL is often mixed with other polymers, including PP, polycarbonate (PC), polyethylene oxide (PEO), and starch, in order to create biocomposites with desired properties. The versatile and biodegradable PCL has been blended with other polymers and considered for use in biomedical applications, such as catheters, blood bags, and packaging [117]. PCL has been degraded by extracellular depolymerase or in a variety of natural environments [118]. PCL has gained popularity as a biodegradable packaging alternative to non-biodegradable products. Despite its many benefits, due to its poor thermal stability, only a few studies have assessed PCL as a biodegradable material for food packaging. PCL’s low melting point makes it easy to process, but it also restricts its applications [119].

Lu et al. (2014) [120] studied the mechanical properties of PCL-hydroxyapatite (HA) porous scaffolds created by Porogen-based solid free form a fabrication method. The ultimate compressive strength of a porous scaffold built of 80/20 polycaprolactone–hydroxyapatite (PCL–HA) composite was 3.7 ± 0.2 MPa, with a compression modulus of 61.4 ± 3.4 Mpa, which is comparable to the compressive strength of the trabecular bone. The compressive characteristics and stiffness of PCL–HA composites were considerably improved (P0.05) when the concentration of HA in the composites was increased. This shows that PCL–HA composites have the potential to be used in bone regeneration. The ultimate tensile strength and tensile modulus of solid PCL and PCL–HA composites increased as the concentration of HA in the composite increased, according to tensile tests.

**Figure 4:**
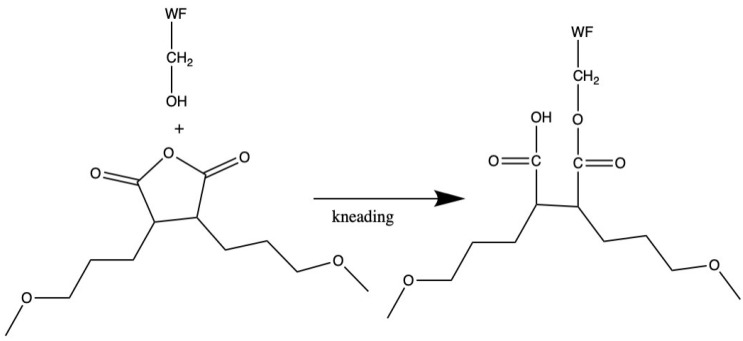
Possible reaction of WF and PCL-g-MA.

Lee et al. (2003) [121] obtained higher tensile modulus from the preparation of wood flour (WF)–PCL biocomposites by kneading process. The PCL grafted maleic anhydride (PCL-g-MA) was used as a compatibilizer. Figure 4 depicts a potential reaction between WF and PCL-g-MA. PCL-g-MA was added to the biocomposites to improve their tensile properties. Tensile strength and Young’s modulus in WF–PCL (50/50, *w*/*w*) biocomposites increased from 13 to 27 MPa and 581 to 1011 MPa, respectively, with the addition of 5% PCL-g-MA. They reported that the addition of PCL-g-MA enhanced the tensile properties. The elongation at break increased from 4 to 7%. These increments on mechanical performance may be related to chemical bonds between the anhydrous group of MA and the OH groups of WF.

Combining a biobased polymeric material, such as wheat gluten (WG), with PCL is one way to cut costs and change material properties. In a study conducted by Finkenstadt et al. (2008) [122], WG was used as a filler in PCL (up to 50% *w*/*w*) to create biodegradable biocomposites. An examination under a microscope revealed a well-distributed particle-matrix system. The tensile strength of the biocomposites decreased linearly as the WG content increased from 0% to 6%. (50% WG). The tensile strength for PCL and WG is between the range of 5 to 15 MPa. Packaging materials, hygiene products, disposable consumer goods, and agricultural products may all benefit from these WG–PCL biocomposites.

In a study on the development of sustainable biodegradable lignocellulosic hemp fiber–PCL biocomposites by Dhakal et al. (2018) [123], it was shown that the biocomposites possess enhanced mechanical properties compared to PCL. The sample was first made by a twin screw extrusion machine with a different l/d fiber ratio of 19, 26, 30, and 38, respectively. The result shows that the tensile strength of biocomposites with higher aspect ratios does not differ much from PCL, apart from the biocomposites with an aspect ratio of 26, although it is significantly weaker in wet conditions compared to dry conditions. However, Young’s modulus of this composite was shown to encourage an increase from 0.14 GPa to 0.40 GPa, signifying a 186% increase from PCL. Based on the result, it is shown that the biocomposites with a fiber aspect ratio of 26 out-performed other samples in both dry and saturated conditions. Since the fiber is inherently hydrophilic, appropriate treatment on surface of fiber is suggested to be carried out to ensure that it is compatible with PCL.

Lakshmipriya et al. (2019) [124] studied the silk–PCL biocomposites using various layers of woven silk fabric as reinforcement, along with the PCL polymer matrix. The stacking of different layers of silk fabric-reinforced PCL biocomposites in this study was prepared using compression molding. The 14 layers of silk–PCL biocomposites showed higher mechanical properties when compared to other composites. This was due to its sufficient mechanical interlocking between the fabric and the matrix. However, there was a slightly decreass in the tensile properties of biocomposites, but it can still be used as a biodegradable plate due to its adequate mechanical strength.

Additionally, several researchers have successfully developed cellulose–PCL biocomposites from various sources of fibers. Theeranattapong et al. (2015) [125] developed the biocomposites of oxidized regenerated cellulose–PCL for use as a synthetic dura meter. Cellulose–PCL biocomposites were prepared with solution infiltration of a cellulose sheet with PCL solution at various concentrations ranging from 10–50 g per 100 mL. It was found that the tensile properties were initially decreased with increasing PCL concentration for up to 20%. Then, it increased with further increasing PCL concentrations. Elongation at break of all formulations was not significantly different. Interestingly, the mechanical properties of the samples were found to be similar to those of natural human dura mater.

Aguiar et al. (2016) [126] prepared a cellulose–PCL biocomposites and evaluated their mechanical performance. In their work, alkaline and acid pre-treatment were performed on fibres to remove the amorphous portion and to aid fibrillation. PCL–cellulose biocomposites were prepared in a twin screw extruder by adding different cellulose (at 2, 5, and 10 wt.%) and maintaining the processing time constant. The results suggest that increasing the cellulose crystallinity leads to an increased nucleation effect for PCL and that cellulose with low crystallinity hinders crystallization of the matrix. Moreover, shorter cellulose fibres promote a higher nucleation effect in the PCL matrix. The dynamic-mechanical analysis showed an increase in the modulus of the biocomposites containing 5 wt.% cellulose.

Moreover, Alemán-Domínguez et al. (2018) [127] manufactured porous cellulose–PCL biocomposites for application as scaffolds. The biocomposites contained 0, 2, and 5 wt.% of the carboxymethyl cellulose. Biocomposites have similar values of porosity and apparent density than the neat PCL. However, biocomposites containing 5 wt.% of carboxymethyl cellulose have micropores on the filaments due to a hindered deposition process. This characteristic affects the mechanical properties of the structures, so these ones have a mean compression modulus significantly lower than the neat PCL scaffolds. However, the mechanical properties of biocomposites containing 2 wt.% of carboxymethyl cellulose show no significant difference with the neat PCL.

Meanwhile, the use of nanocellulose in PCL-based biocomposites has also been reported in several studies. Sheng et al. (2014) [128] investigated the mechanical performance of electro-spun PCL–CNC biocomposite mats. The results showed that the mechanical properties of the biocomposite mats were improved and that the surface hydrophilicity was increased in comparison with neat PCL mats. The tensile strength of the biocomposites mats was improved following an increase in CNC loading. The maximum strength appeared at 10 wt.% CNC loading, and then it gradually decreased. This is due to the higher CNC content which led to the aggregation of CNC. Moreover, the modulus was significantly increased compared to the neat PCL mat. However, the strain at break decreased as compared to the neat PCL.

Table 7 summarizes the mechanical properties of several PCL-based green biocomposites.

### 4.2. Thermal Properties of Polycaprolactone-Based Green Biocomposites

There are several studies which have reported on the thermal properties of PCL-based green biocomposites. In 2014, Khandanlou et al. [135] investigated the effect of modified rice straw (RS) on the thermal properties of PCL biocomposites prepared via the solution–casting method. The concentration of rice straw was varied. Based on the thermal properties analysis, the TGA thermograms revealed that the biocomposites have a lower on-set temperature for thermal degradation than neat PCL. The TGA and DTG measurements of degradation temperature at 5.0, 10.0, 50.0, and 80.0 percent fibers degradation are shown in Table 8.

**Figure 5:**
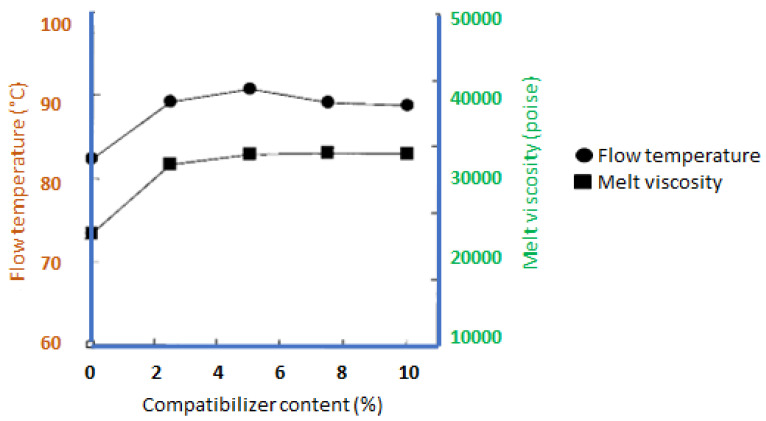
Effects of PCL-g-MA content on the thermal properties of WF–PCL biocomposites [121].

On the other hand, Lee et al. [121] has studied the thermal flow properties of wood flour (WF)–PCL biocomposites. As a compatibilizer, PCL-g-MA was used in this study. The effects of the compatibilizer material on the flow properties of the WF–PCL biocomposites are shown in Figure 5. It shows that adding 2% PCL-g-MA increased the melt viscosity from 11,600 to 27,100 poise and increased the thermal flow temperature from 82 °C to 89 °C. During the kneading reaction, esterification occurred between the acid anhydride groups of PCL-g-MA and the OH groups of WF, as shown by the variations in flow temperature and melt viscosity. When MA and WF are mixed and heated to more than 60 °C, esterification easily occurs without the use of a catalyst or a solvent [136].

## 5. Polycaprolactone-Based Hydrid Biocomposites

Over the past few years, there has been continuous improvement in the performance of PCL by producing hybrid biocomposites. PCL hybrid biocomposites have been developed by various researchers, combining PCL with several other compounds, such as acrylic acid, epoxy, unsaturated polyester, phenolic, vinyl ester, as well as natural fibers [137,138,139,140,141,142,143]. In this section, the mechanical and thermal properties of PCL hybrid biocomposites are presented.

### 5.1. Mechanical Properties of Polycaprolactone Hybrid Biocomposites

The hybridization of biocomposites material is the key to designing new components with good strength at a relatively lower cost [110]. The mechanical properties of PCL hybrid biocomposites gives an advantage of good strength at a lower cost, which can be used for applications that were not possible by using the green biocomposites [144,145,146].

Wu et al. (2010) [147] investigated the structural and thermal effects of substituting a more compatible acrylic acid-grafted PCL (PCL-g-AA) for neat PCL in coconut fibre (CF) containing biocomposites. CF is commonly available as a by-product of coconut production in Taiwan’s coastal regions. A coconut weighs about 600 g and contains drinkable juice between the green outer shell and the hard nut in the centre. Within the coconut, there is a 3–5-cm-thick white fibrous content. In their study, the formation of branched and cross-linked macromolecules through reactions between carboxyl groups in PCL-g-AA and hydroxyl groups in CF resulted in significantly more homogeneous fibre dispersion in the PCL-g-AA matrix. The PCL-g-AA–CF biocomposites had a significantly higher tensile strength at break than he tPCL–CF biocomposites. Due to its lower melt viscosity, the PCL-g-AA–CF blend was also easier to process. From the study, it shows that the tensile strength of PCL–CF can range between 12 to 30 MPa.

The combination of strength and elasticity has been a particular challenge in the development of synthetic grafts, with conventional materials, such as segmented polyurethanes, which can be modified to strengthen one at the expense of the other [148]. Wise et al. (2011) [149] conducted study on multilayered synthetic human elastin/PCL hybrid vascular graft with tailored mechanical properties. The result shows that the elastin–PCL hybrid biocomposites have the lowest tensile strength within a range of 500–520 kPa due to its specific requirement. Tropoelastin was then added to a synthetic elastin–PCL conduit with mechanical properties similar to the human internal mammary artery, including permeability, compliance, elastic modulus, and burst strain. This multi-layered conduit also demonstrated suturability and mechanical durability in a small-scale rabbit carotid interposition model by presenting a synthetic elastin internal lamina to circulating blood. According to the researchers, synthetic elastin was successfully engineered into a completely synthetic vascular graft that matches the mechanical properties of the internal mammary artery.

Moreover, several other research have reported on the mechanical properties of cellulose/PCL hybrid biocomposites. Ali (2016) [150] prepared hybrid biocomposites of cellulose acetate propionate (CAP)–starch–PCL for packaging applications. CAP was used as a cellulose derivative to enhance the physical properties of biocomposites. The addition of CAP to starch and PCL blends improved the mechanical properties, even at a high content of starch. It was found that the tensile strength and elongation at break of CAP–starch–PCL biocomposites were increased. It is suggested that CAP improved the compatibility and the interfacial adhesion between PCL and starch. Moreover, it was found that the tensile yield strengths of most CAP–starch–PCL biocomposites were higher than the yield tensile strength of neat PCL. They suggested that it may be due to the existence of an interaction between this hybrid components.

Yu et al. (2018) [151] developed a graphene oxide–CNC–PCL biocomposite using quaternary ammonium salt systems to be used in ultraviolet-shielding films. The preparation of biocomposites involved grafting the CNC with PCL by ring-opening copolymerization catalysed by 4-dimethylaminopyridine in a dual tetrabutylammonium acetate–dimethyl sulfoxide solvent system. Then, a novel ultraviolet-shielding film based on CNC–PCL biocomposites was prepared by introducing graphene oxide. The results obtained showed that the introduction of graphene oxide not only obviously influenced the inherent structure of the hybrid biocomposites but remarkably changed the surface morphology of the film. Moreover, the graphene oxide–CNC–PCL hybrid biocomposites showed a significant improvement in tensile strength, from 2.63 to 4.55 MPa, as well as elongation-at-break, from 6.4 to 15.5%, compared with the CNC–PCL biocomposites, owing to the strong hydrogen-bonding interaction that physically crosslinked graphene oxide with CNC–PCL biocomposites.

Gibril et al. (2019) [152] studied on the effect CNC and zinc oxide (ZnO) to the mechanical properties of PCL hybrid biocompsites. CNC loadings at 2–8 wt.% and ZnO were added onto the PCL matrix. Increasing CNC–ZnO content enhances the tensile strength with low filler contents at 2 and 4 wt.%, due to good dispersion of the CNC within the matrix, which yields a higher reinforcement effect. However, higher CNC loadings at 6 and 8 wt.% loading resulted in a gradual decrease in the tensile strength of hybrid biocomposites due to the agglomeration of the CNC, which restrains stress movement, interrupts the matrix segmental mobility, and promotes rigidity. By adding different amounts of CNC, the elongation at break of hybrid biocomposites decreased (at loading of 6 and 8 wt.%), except for samples prepared with low content of CNC (2 and 4 wt.%). This also influenced by the dispersion of CNC within the PCL matrix.

Recently, Quilez-Molina et al. (2021) [153] developed curcumin-functionalized cellulose–magnesium and carbonate–PCL hybrid biocomposites. Magnesium carbonate (MgCO_3_) was used as a food additive. Meanwhile, cellulose was functionalized with ethanoic curcumin solutions to protect them against oxidative degradation. The development of this material is simple, cost-effective, non-toxic, and paper-like. Flexible bio-composites can be fabricated on large scale and can be used in several applications, ranging from sustainable packaging to medical applications and freshness indicators. They reported that the mechanical properties of hybrid biocomposites depend on different factors, such as the fiber direction, nature of the fibers, length, and the bonding strength between the fibers and polymers. It was found that functionalizing the cellulose with curcumin did not affect the mechanical properties of hybrid biocomposites. Moreover, the warp direction of hybrid biocomposites exhibited higher elastic modulus (ten times more than that of the weft direction).

Table 9 summarized the mechanical properties of several PCL-based hybrid biocomposites.

### 5.2. Thermal Properties of Polycaprolactone-Based Hybrid Biocomposites

The thermal stability of the hybrid biocomposites can be significantly improved as compared to the neat polymer. In recent years, there has been a lot of research to improve the thermal properties of hybrid biocomposites [110,155]. The properties of the hybrid biocomposites have been enhanced by the addition of nanomaterials, metallic, carbon, and ceramic-based fillers.

Hybrid biocomposites have comparable strength and properties to many traditional plastics as a result of the inclusion of nanoclay. Furthermore, the use of natural fibers in hybrid biocomposites lowers the cost of biodegradable polymer, expanding the range of applications for biodegradable polymer-based biocomposites. The applications of hybrid biocomposites may be more suitable for single-use materials that do not require a lot of strength, though biodegradability and environmental friendliness are also important considerations in material selection.

Abdolmohammadi et al. (2011) [156] investigated the effect of thermal properties of PCL/chitosan hybrid biocomposites when sodium montmorillonite (Na MMT) and octadecylamine montmorillonite (ODA-MMT) are used. Based on the result obtained, the degradation temperature is increased in both micro and nanocomposites when Na MMT and ODA-MMT are added to the PCL/chitosan blend, with the maximum thermal stability observed at 1 wt.% Na MMT and 3 wt.% ODA-MMT loadings. The degradation becomes faster at higher ODA-MMT loadings than 3 wt.% due to the higher amount of modifier in the matrix. Since the modifier has a lower thermal stability, it degrades more quickly. Besides that, by loading 1 wt. 100% Na MMT and 3 wt.% ODA-MMT, the onset temperature of PCL/chitosan biocomposite increases from 231.45 °C to 238.76 °C and 254.71 °C, respectively. Two stages of degradation of PCL/chitosan blend was observed in the experiment. The first step, which occurs at T_max1_ = 283 °C, is chitosan backbone degradation caused by saccharide rings dehydration, and the second stage, which occurs at T_max2_ = 382 °C, is PCL decomposition. After adding 1 wt.% Na MMT and 3 wt.% ODA-MMT, the second stage (T_max2_) rises significantly from 382.89 °C for the PCL/chitosan biocomposite to 392.55 °C and 412.72 °C, respectively. The increase in degradation temperature is due to the clay’s high thermal stability, which limits heat transmission and decreases the diffusion of volatile substances released by substances. Due to its exfoliated-intercalated structure and improved interaction between ODA-MMT and matrix, nanocomposites have a higher thermal stability than micro composites. As a result, thermal stability in nanocomposite and microcomposite materials has improved, especially in nanocomposites, where the maximum thermal stability was observed at 3 wt.% ODA-MMT loading. The broken surface morphology of the PCL–chitosan blend becomes more stretched and homogeneous in PCL–chitosan–ODA-MMT than in PCL–chitosan–Na MMT.

Moreover, Eng et al. (2014) [157] investigated the effect of adding 1 wt.% hydrophilic nanoclay on PLA–PCL–oil palm mesocarp fiber (OPMF) biocomposites towards the thermal properties. Previous study stated that adding nanoclay to PLA–PCL–OPMF biocomposites improves their thermal stability. The PLA–PCL–OPMF biocomposites have an onset temperature of 196.01 °C, which increases to 221.12 °C when clay is added. Table 10 shows the thermal properties of PLA–PCL–OPMF biocomposites and PLA–PCL/1 wt.% clay/OPMF hybrid biocomposites. By increasing the thermal stability of hybrid biocomposites, clay will minimize the permeability of volatile degradation materials. The dispersed clay, in contrast to the smooth polymer, acts as a barrier, delaying the release of thermal degradation products.

## 6. Applications of Polycaprolactone-Based Biocomposites

PCL has been widely applied in biomedical fields, especially in tissue engineering and medical implants, because they are biodegradable and have high biocompatibility. Manivasagam et al. (2019) [23] mentioned that polycaprolactones are being explored extensively in bone tissue engineering due to a lack of bioactivity and high degradation rates. Miller et al. (2011) [20] have stated that PCL can potentially be used as a bioscaffold. Nevertheless, when it comes to the application of tissue engineering, PCL suffers from some shortcomings, such as poor mechanical properties, slow degradation rate, and low cell adhesion. The reinforcement of bioactive glasses and calcium phosphate-based ceramics within PCL has created a class of hybrid biomaterials with improved controllable degradation rates, good mechanical properties, and enhanced bioactivity, making them suitable for bone tissue engineering [158]. PCL green biocomposites with superior mechanical properties can potentially be used as orthopaedic implants, as briefly outlined as.

The Food and Medication Administration (FDA) has approved PCL for use in specified applications in the human body as a suture, drug delivery device, or adhesion barrier [8,159,160,161]. Following the recent launch of a PCL-based microsphere dermal filler belonging to the collagen stimulator class (Ellansé), PCL is being employed in the field of human aesthetics [162,163]. PCL-based products have also been utilized to treat facial ageing indications, such as contour laxity and volume loss, by stimulating collagen formation, resulting in an immediate and long-lasting natural impact [159,163,164,165,166,167]. In addition, it is being studied as a scaffold for tissue engineering-mediated injury repair using the guided bone regeneration (GBR) membrane, which has been extensively reported by numerous researchers [168,169,170,171,172,173,174]. It has been utilized as a hydrophobic block in amphiphilic synthetic block copolymers that are used to build the vesicle membrane of polymersomes [175,176].

**Figure 6:**
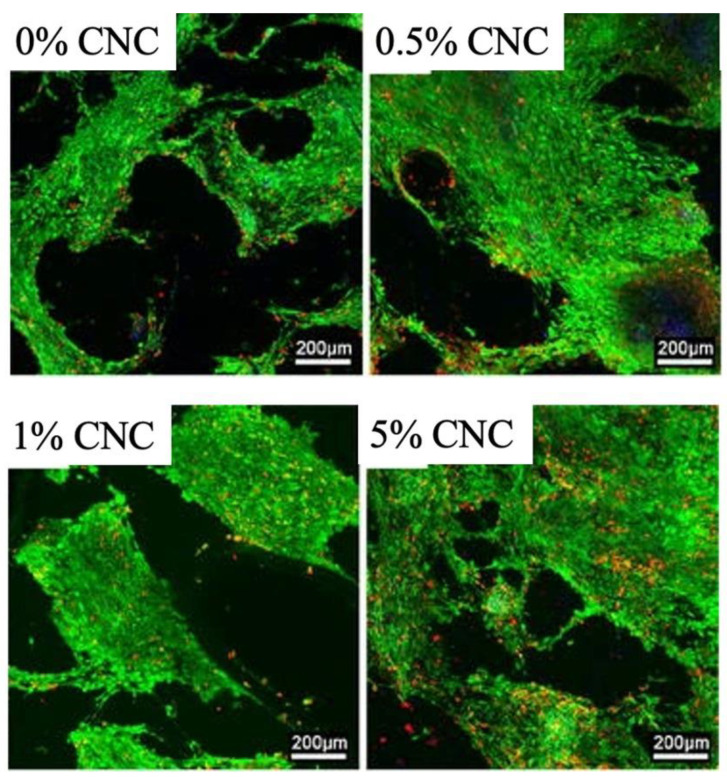
Florescence images with a scale bar of 200 μm of 3T3 fibroblast cell culture results at day 10 of microcellular injection moulded samples: neat PCL, 0.5% CNC, 1% CNC, and 5% CNC. Adapted with copyright permission from ref. [178].

Recently, PCL green biocomposites have been developed and commercialized. The majority of PCL green biocomposites are currently in the research and development stage. To make green biocomposites at a cheaper cost, new processing techniques and technologies are being developed [177]. Hao and co-workers found that the twin screw extrusion was used to make PCL–CNC nanocomposites. Microcellular nanocomposite samples were created utilising microcellular injection moulding and a physical blowing agent of carbon dioxide (CO_2_). The biocompatibility of the material was examined. The result is depicted in Figure 6. The green hue in the florescence photographs symbolises live cells, whereas the red colour depicts dead cells. In comparison to bare dead cells, the clean PCL sample exhibits a substantial number of living cells. It was also found that 0.5% CNC and 1% CNC samples had mostly live cells, thus indicating good compatibility between the substrate and the cells.

**Figure 7:**
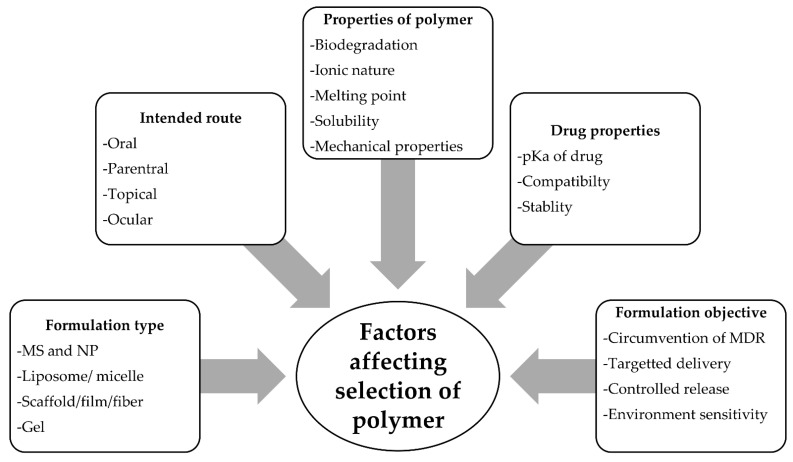
Factors affecting polymer selection.

**Figure 8:**
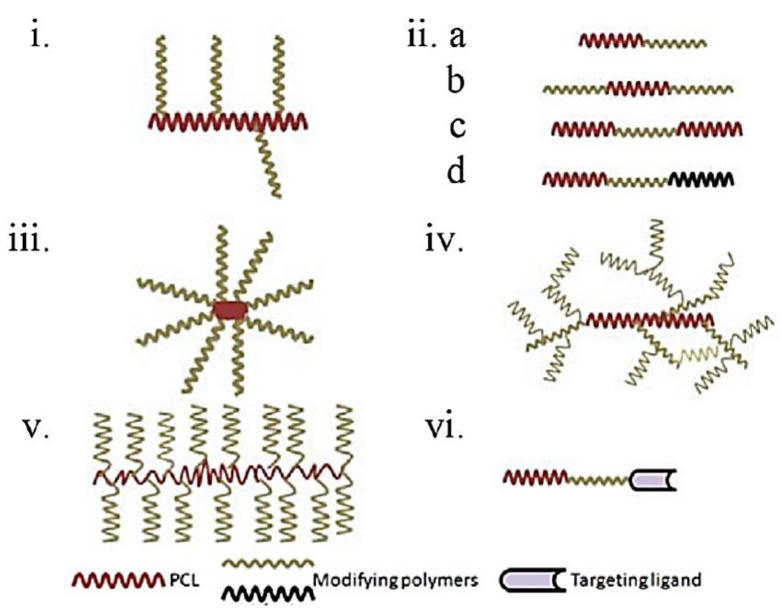
Different demonstrated architecture of PCL modification: (**i**) graft copolymer; (**iia**) diblock copolymer; (**iib**,**c**) triblock copolymer; (**iid**) tricomponent triblock copolymer; (**iii**) sta- shaped copolymer; (**iv**) hyperbranched copolymer; (**v**) molecular brush or comb shaped copolymer; and (**vi**) targeted block copolymer. Adapted with copyright permission from ref. [190].

PCL beads have been used to encapsulate a range of medicines for controlled re-release and targeted drug delivery [179,180,181,182,183,184,185,186,187,188,189]. In addition to polymeric properties several other factors, such as the type and objective of formulation, the route of administration, drug or polymeric properties etc., also affect the selection of the polymer (Figure 7). Hence, selection of a polymer is an important step for the development of a successful drug delivery system or device [190]. PCL-based modified polymers demonstrated so far predominantly involve its copolymers with several other polymers in different forms (Figure 8). Nevertheless, other modifications (blends and composite) were also reported in different formulations [191,192].

Among biodegradable polymers modified for amelioration of properties, a special focus is made on PCL mainly due to its broad spectrum of compatibility with a wide range of other polymers. Its versatile nature, ease of fabrication, and biocompatibility establish it to be the polymer of interest by investigators worldwide for drug delivery and tissue engineering applications [193,194,195,196,197]. However, when we consider properties of unmodified PCL, there are considerable restrictions for its use. For example, its hydrophobic nature does not allow the facile release of hydrophobic drugs (from prepared formulations) and micelle formation. Furthermore, long-term degradation (ranging from weeks to months) slows down tissue replacement in the case of scaffolds, mechanical property limits its application to hard tissue engineering only, and nonreactivity is unsuitable for preparation of NC. Therefore, attempts have been made to overcome these undesirable properties by various types of modification, as mentioned in Figure 8, for successful application in pharmaceutical formulations. To great excitement, the use of modified PCL dominated over the past decade by virtue of which PCL was demonstrated in almost all novel formulations overcoming the above-mentioned restrictions. Additionally, functionalization as a result of PCL modifications is a featured advantage and is considered as another cause for this typical preference for modified PCL.

**Figure 9:**
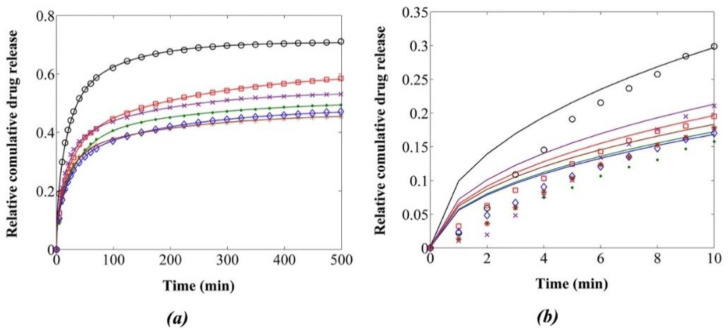
Drug release diagram of drug loaded samples with various concentration of CNC (Experimental data for ○ PCL, □ PCL-CNC0.5%, × PCL-CNC1.0%, ● PCL-CNC1.5%, ▯ PCL-CNC2.5%, ◊ PCL-CNC4%. Reproduced from ref. [199].

Additionally, the possibility of using PCL to be used as implants for targeted drug delivery has been explored. Boia et al. (2019) [198] have developed a novel way of using porous polycaprolactone as an intraocular implant to deliver dexamethasone to replace eye drops or intravitreal injections. The implants were made by using green supercritical carbon dioxide foaming or mixing methods, resulting in the implant having high porosity and high surface area. This, in turn, will cause a higher degradation rate than typical PCL-based implants, which have a relatively slower degradation rate. They are then inserted into adult rats for further observation. The implant is shown to have good biocompatibility, since it does not cause cell death or reduce the number of neurons [198]. In contrast, Hivechi et al. (2019) [199] investigated the regulated release of tetracycline hydrochloride using CNC-reinforced PCL nanofibers. The amount of CNC in the PCL nanofibers was increased, which resulted in a delayed release of a medication, as depicted in Figure 9a,b.

## 7. Challenges and Opportunities

PCL is a unique and versatile biomaterial equipped with several importance features, including ease of processability and good stability under ambient conditions. In fact, this biomaterial has been reported to be approved by U.S Food and Drug Administration for use in several products [200]. Nevertheless, with the great potential of PCL biocomposites and its combination of desirable properties, it is deemed that this versatile biomaterial is not fully translated into a wide area of applications, especially medical sectors, such as tissue engineering and clinical application. This situation is due to several factors associated with PCL. The several challenges faced by PCL biocomposites are discussed in this section, together with its opportunities for future direction.

The main concern of neat PCL is that it is not suitable for some of tissue engineering, especially bone tissue engineering, due to the degradation and mechanical properties of this material. The most effective approach is to use it as one of the elements of blend materials. On the other hand, the incorporation of PCL with several available nanostructured filler such as nanocellulose could be a vital strategy to enhance the PCL biocomposites for its degradation and mechanical properties [201].

On the other hand, due to its hydrophobicity and insufficient wettability, PCL exhibits poor cell attachment [202]. Thus, in order to overcome this hurdle, surface functionalization is deemed to be the best approach to improve the feature of PCL biocomposites. In reviews, several efforts have been demonstrated to modify the surface of this biomaterial. Zander et al. (2010) [203] have demonstrated the functionalization of aligned and unaligned PCL electro-spun fibers using physical adsorption. The extracellular matrix proteins laminin and collagen have been utilized, resulting in the enhancement of cell attachment and further increasing neurite outgrowth. In another report, surface-initiated atom transfer radical polymerization (ATRP) has been demonstrated for its application in functionalized PCL film surfaces [204]. The results showed improved cell adhesion properties of PCL and further enhanced the cell proliferation on the incorporated collagen functionalized PCL. This finding is deemed as an important discovery to nanocomposite industries, especially for PCL-based biomaterials with widened applications in biomedical sector.

All in all, the tremendous efforts have been proposed to improve PCL-based biocomposites. With the rise in these challenges, a new path of improvement has been increasingly beneficial in various sectors.

## 8. Conclusions

PCL is truly a promising materiel for the future to replace the current materials that are impossible and expensive to reuse. The aim of this review is to investigate the potential of PCL biocomposites reinforced with natural fibres to enhance the quality of the produced biocomposites. Blending and processing of PCL into biocomposites has improved the materials’ properties, thus providing more area to exploit these excellent properties. In this review, green biocomposites and hybrid biocomposites of PCL were evaluated in terms of their mechanical and thermal properties. The characteristics and properties of natural fibres can greatly influence the final properties of PCL-based biocomposites. Most of the studies reported that the performance of the PCL biocomposites improved, as compared to the neat PCL after blended with natural fibres. Moreover, emerging uses of nanofillers, such as nanocellulose and MMT, also managed to greatly improve the performance of PCL-based green and hybrid biocomposites. Besides that, PC-based biocomposites have huge utilization and potential within drug delivery devices, medical devices, and tissue engineering.

## Figures and Tables

**Table 1:** List of biopolymers.

Type of Biopolymer		Example
Natural biopolymer	Plant carbohydrate and animal	Starch, cellulose, nanocellulose, agar, chitosan, etc.
	Plant origin protein	Soy protein, corn zein, wheat gluten, gelatine, collagen, whey protein, casein
Synthetic biodegradable polymer		Poly(L-lactide), polyglycolic acid, polycaprolactone, polybutylene succinate, polyvinyl alcohol, etc.
Biopolymers produced by microbial fermentation		Polyhydroxyalkanoates including poly-β-hydroxybutyrate, poly-3- hydroxybutyrate-co-3-hydroxyvalerate, etc.

**Table 2:** Properties of PCL with different molecular weights.

Molecular Weight	Melting Point, °C	Tensile Stress, N/m^2^	Elongation at Break, %
37,000	58–60	1.37 × 10^7^	660
50,000	58–60	3.53 × 10^7^	800
80,000	60–62	5.69 × 10^7^	900

**Table 3:** The degradation behaviour of the biodegradable polyesters. Reproduce from ref. [24].

Polyester	Degradation By-Products (pKa)	In Vivo Degradation Rate	Degradation Mechanism
PCL	Caproic acid (4.88)	50% in 4 years 1% in 6 months	Hydrolytic degradation
PLA	Lactic acid (3.85) Lactic acid (3.08)	50% in 1–2 years 98% in 12 months 100% in >12 months 100% in 12–16 month	Hydrolysis through the action of enzymes
PGA	Glycolic acid (3.83)	100% in 2–3 month 100% in 6–12 months	Both enzymatic and non-enzymatic hydrolysis

**Table 4:** Advantages and disadvantages of PCL.

Advantages	Disadvantages
High biocompatibility	Adheres poorly to cells
Highly biodegradable	Toxic solvent
Great electrospinning properties	Low melting point
Long biodegradable time	Complex and expensive production
High material purity	

**Table 5:** Chemical composition of selected fibers.

Fibers	Chemical Compositions (wt.%) *			
	Cellulose	Hemicellulose	Lignin	Pectin
Abaca	62.5	21	12	0.8
Alfa	45.4	38.5	14.9	-
Bagasse	37	21	22	10
Banana	62.5	12.5	7.5	4
Bamboo	34.5	20.5	26	-
Coir	46	0.3	45	4
Cotton	89	4	0.75	6
Curaua	73.6	5	7.5	-
Flax	70.5	16.5	2.5	0.9
Hemp	81	20	4	0.9
Henequen	60	28	8	-
Isora	74	-	23	-
Jute	67	16	9	0.2
Kapok	13.16	-	-	-
Kenaf	53.5	21	17	2
Nettle	86	10	-	-
Phormium	67	30	11	-
Piassava	28.6	25.8	45	-
Pineapple	80.5	17.5	8.3	4
Ramie	72	14	0.8	1.95
Sisal	60	11.5	8	1.2

* Note that the total composition in some fibers may not add up to 100% due to presence of pectin, lignin and waxes, which may exist in negligible amount and can be considered as zero.

**Table 6:** Mechanical properties of natural fibers.

Type of Fiber	Diameter (μm)	Density (g/cm^3^)	Tensile Strength (MPa)	Young’s Modulus (GPa)
Abaca	250–300	1.5	717	18.6
Jute	250–2500	1.3–1.49	393–800	13–26.5
Sisal	205–230	1.41	350–370	12.8
Kenaf	83.5	1.2	282.60	7.13
Coconut	396.98	1.2	140–225	3–5
Bamboo	-	1.2–1.5	500–575	27–40
Date palm	-	0.463	125–200	-
Banana	-	0.95–0.75	180–430	-
Reed	-	0.49	70–140	-

**Table 7:** Mechanical properties of PCL-based green biocomposites.

Green Biocomposites	Processing Technique	Mechanical Properties				Ref.
		Tensile Strength	Tensile Modulus	Flexural Strength	Flexural Modulus	
Cellulose acetate–PCL	Solvent casting	10 MPa	-	400 MPa	-	[129]
Cellulose–PCL	Solution infiltration	2.8–6.2 MPa	15–44 MPa	-	-	[125]
Cellulose–PCL	Extrusion	2.12 ± 0.15 to 2.35 ± 0.51 MPa	-	-	18.7 ± 3.1 to 12.5 ± 4.0 MPa	[127]
CNC–PCL	Electrospinning	1.1–1.6 MPa	7.2 MPa	-	-	[128]
Wood flour–PCL	Knead processing	13–27 MPa	581–1011 MPa	-	-	[121]
Wheat–PCL	Blending	5–15 MPa	160–500 MPa	-	-	[122]
Starch–PCL	Predrying of starch	29.49 ± 2.7 MPa				[130]
Silk fabric–PCL	Compression moulding	92.93 ± 3.273 MPa	1.143 ± 0.108 MPa	36.036 ± 1.903 MPa	2.688 ± 0.0124 MPa	[124]
HAP–PCL	Grafted and blended techniques	2.53 ± 0.21 MPa	111.92 ± 3.97 MPa	-	-	[131]
Cellulose–PCL	Wet feeding	31 ± 0.71 MPa	1.85 ± 0.08 MPa	-	-	[132]
Cellulose–PCL	Dry-feeding	24 ± 1.24 MPa	0.59 ± 0.06 MPa	-	-	[132]
Cellulose–PCL	Electrospinning process	4.45 ± 0.32 MPa	19.17 ± 0.8 MPa	-	-	[133]
Oil palm Fiber–PCL	Melt blending	9.8 MPa	250 MPa	-	-	[134]

**Table 8:** TGA and DTG measurements of degradation temperature at 5.0, 10.0, 50.0, and 80.0 percent fiber degradation. Reproduce with copyright permission from Khandanlou et al. [135].

Sample	T5% (°C)	T10% (°C)	T80% (°C)	Tmax (°C)	T5% (°C)	Residue at 500 °C (%)
ORS	222.55	343.11	452.60	452.60	404.45	17.2
PCL	380.01	404.16	418.50	418.50	409.04	5.0
1.0%	367.95	400.57	413.62	413.62	402.27	5.2
3.0%	356.29	393.07	409.08	409.08	400.10	6.3
5.0%	349.94	388.53	404.37	404.37	387.76	8.1
7.0%	343.13	381.72	400.15	400.15	372.95	10.9

**Table 9:** Mechanical properties of PCL hybrid biocomposites.

Hybrid Biocomposites	Processing Technique	Mechanical Properties				Ref.
		Tensile Strength	Tensile Modulus	Flexural Strength	Flexural Modulus	
CAP–starch–PCL	Internal blending	Increased (value is not reported)	Increased (value is not reported)	-	-	[150]
Graphene oxide–CNC–PCL	Solvent casting and ultrasonication	4.55 ± 0.15 MPa	-	-	-	[151]
Zinc oxide–CNC–PCL	Solvent casting	12.16 ± 0.51 to 15.41 ± 0.79 MPa	61.71 ± 0.17 to 91.71 ± 0.23 MPa	-	-	[152]
Elastic–PCL	-	500–520 kPa	257–281 kPa	-	-	[149]
Gelatin–acetylated CNF–PCL	Electrospun	2.5–4.3 MPa	21.3–24.1 MPa	-	-	[142]
Coconut–acrylic acid–PCL	Blending	12–30 MPa	-	-	-	[147]
Alginate–PCL(electrospun)–PCL(struts)	Electrospun	0.3–12.5 MPa	0.9–10 MPa	-	-	[154]

**Table 10:** Thermal properties of PLA–PCL–OPMF biocomposites and PLA–PCL/1 wt.% clay/OPMF hybrid biocomposites.

Sample	Onset Temperature (°C)	Offset Temperature (°C)	Percentage of Degradation
PLA–PCL–OPMF	196.01	473.84	72.78
PLA–PCL/1 wt.% clay–OPMF	221.12	458.36	71.08

## Data Availability

Not applicable.
